# Drop drying on surfaces determines chemical reactivity - the specific case of immobilization of oligonucleotides on microarrays

**DOI:** 10.1186/2046-1682-6-8

**Published:** 2013-06-12

**Authors:** Jens Sobek, Catharine Aquino, Wilfried Weigel, Ralph Schlapbach

**Affiliations:** 1 Functional Genomics Center Zurich, ETH Zurich/ University of Zurich, Winterthurerstrasse 190, Zurich, CH-8057, Switzerland; 2 Institute of Chemistry, Humboldt University Berlin, Brook-Taylor-Strasse 2, Berlin, 12489, Germany

## Abstract

**Background:**

Drop drying is a key factor in a wide range of technical applications, including spotted microarrays. The applied nL liquid volume provides specific reaction conditions for the immobilization of probe molecules to a chemically modified surface.

**Results:**

We investigated the influence of nL and μL liquid drop volumes on the process of probe immobilization and compare the results obtained to the situation in liquid solution. In our data, we observe a strong relationship between drop drying effects on immobilization and surface chemistry. In this work, we present results on the immobilization of dye labeled 20mer oligonucleotides with and without an activating 5′-aminoheptyl linker onto a 2D epoxysilane and a 3D NHS activated hydrogel surface.

**Conclusions:**

Our experiments identified two basic processes determining immobilization. First, the rate of drop drying that depends on the drop volume and the ambient relative humidity. Oligonucleotides in a dried spot react unspecifically with the surface and long reaction times are needed. 3D hydrogel surfaces allow for immobilization in a liquid environment under diffusive conditions. Here, oligonucleotide immobilization is much faster and a specific reaction with the reactive linker group is observed. Second, the effect of increasing probe concentration as a result of drop drying. On a 3D hydrogel, the increasing concentration of probe molecules in nL spotting volumes accelerates immobilization dramatically. In case of μL volumes, immobilization depends on whether the drop is allowed to dry completely. At non-drying conditions, very limited immobilization is observed due to the low oligonucleotide concentration used in microarray spotting solutions. The results of our study provide a general guideline for microarray assay development. They allow for the initial definition and further optimization of reaction conditions for the immobilization of oligonucleotides and other probe molecule classes to different surfaces in dependence of the applied spotting and reaction volume.

## Background

Microarray technology has become the platform of choice for the high-throughput analysis of a variety of substrate classes including DNA, proteins, peptides and glycans. In particular, oligonucleotide microarrays have become an established tool to study e.g. gene expression profiling, SNP genotyping, and enzymatic reactions [1-7]. Although there is a wealth of studies investigating nearly every aspect of DNA microarray production, properties, and processing [8-15], little attention has been paid so far to the fact that the special conditions provided during drop drying processes have a crucial influence on the immobilization and further performance of oligonucleotides as probe molecules.

Immobilization is a critical step in microarray processing [16-18]. It not only determines the amount of probe molecules bound to the surface, but also their molecular conformation that provides the basis for molecular recognition in binding experiments [19-21]. Basically, immobilization depends on three factors: the reactivity of the probe, the properties of the slide surface, and the ambient conditions. Drop drying on the other hand, is a fundamental process contributing to such diverse phenomena and applications like cloud formation, fuel evaporation in combustion engines, ink-jet printing, and many others [22]. On spotted microarrays, drop drying determines the time that probe molecules are located in a liquid environment and leads to increasing concentrations of probe and buffer components over time. Although very important, to our knowledge, so far no systematic investigation of drop drying effects on probe immobilization on microarrays has been published. In the specific case of widely used nL spotting, the fast drying of the spotted drop provides special reaction conditions on the surface. In this publication we demonstrate that the interplay between the three mentioned factors is crucial for oligonucleotide immobilization.

In order to compensate for the low reactivity of unmodified oligonucleotides, an activating linker is often used to increase their binding efficiency to the solid support. Besides facilitating immobilization, the linker increases the distance of the immobilized probe to the surface and thereby the conformational flexibility, which leads to a stronger binding of target molecules. Vainrub has shown in a theoretical study that a close proximity of the probe molecule to the surface has an effect on the equilibrium constant and the melting temperature of immobilized oligonucleotides compared to liquid phase hybridization [23]. This work was experimentally confirmed by a number of groups who showed that increasing the distance of the immobilized probe from the surface significantly increases the yield of hybridization [24-26]. In practice, a linker consists of a flexible chain (often alkyl or oligoethylene glycol) and a reactive functional group (often nucleophilic as amino or thiol) attached at one end of the oligonucleotide [17,27-31]. However, theory demands a linker size in molecular dimensions similar to the oligonucleotide. Hexyl, heptyl or dodecyl alkyl chains are too short for this purpose although widely used with oligonucleotides. To fulfill the theoretical requirements, in various studies long sequences of thymine were added to oligonucleotides [25,32,33]. Still, the mere presence of an activating linker does not guarantee an end-linked oligonucleotide immobilization. As we show in the following, immobilization via the linker has to compete with the chemical reactivity of other molecular sites and requires very specific experimental conditions to be efficient that often are not given in a fast drying drop. As a consequence, a reactive linker may be found to have no effect on immobilization in practice. Based on these considerations, in the present study we focus on the effects of a reactive primary amine group on immobilization efficiency, for which the use of a short aminoheptyl linker is sufficient.

Another frequently underestimated experimental factor is the surface architecture, which affects the immobilization efficiency by facilitating immobilization or hindering it by interfering with diffusion and reorientation processes of the probe molecules. Chemical reactions at surfaces are subject to restrictions including sterical hindrance, smaller diffusion coefficients, and changed reactivity compared to reaction in solution [23,24,30,34-36]. As we show for the specific case of oligonucleotides, immobilization is further influenced by the drying rate of the spotted solution, making the volume of the applied drop and the ambient conditions key factors determining the immobilization process. In a typical microarray experiment (sub-) nL drops are delivered from a spotting device. In the early days of array production, when spotting technology was not generally available, common pipettes were used to apply μL drop volumes [25,37]. Later, protocols established from μL experiments were transferred to microarrays generated by (sub-) nL spotting. Wrong conclusions were drawn with respect to the necessity of an activating amine linker since the early experiments were performed under non-drying drop immobilization conditions (liquid phase in larger volumes). To clarify inconsistent reports, we investigated the immobilization process in nL and μL drops, as well as in liquid solution. The latter plays an important role in many current technologies and applications, including the immobilization of oligonucleotides on nanoparticles [38-40], microspheres [41], magnetic microparticles [42], polymer [43,44] and glass beads [45] for microarraying [42] and flow cytometry [46], surface plasmon resonance [47], and next generation sequencing (Roche 454, ABI SOLiD, Ion Torrent and Illumina).

For our experiments, we used epoxysilane and NHS activated hydrogel slides that are both widely used microarray substrates: high quality borosilicate glass coated with 3-glycidoxypropyl-trimethoxysilane (referred to as 2D epoxy) is a typical 2D surface that has become a standard for oligonucleotide applications. Commonly prepared from liquid solution, the silane forms a nm thick polysiloxane network covalently attached to the surface [48]. The surface-bound silane epoxy groups are sufficiently stable during the spotting procedure that can take up to several hours. Epoxysilane slides are easy to process since there is no additional chemical reaction required to achieve a stable covalent immobilization. Spotting oligonucleotides in a variety of different buffers and within a range of concentrations results in arrays with good spot morphology, which is a key factor and a precondition for high quality data and low experimental error [49,50]. A second type of well-established surface coating consists of a thin layer of a hydrogel, typically carrying NHS functionalities. Originally developed for oligonucleotide applications, hydrogel slides have become a useful support particularly for antibody microarrays that make use of the non-drying conditions within the gel [51-53]. A hydrogel consist of a three-dimensional polymer network created by surface-initiated polymerization and subsequent crosslinking or by spin coating of a polymer. The thickness of hydrogel layers on microarray slide surfaces are reported to be in the nm to μm range [54-56]. These slides typically have a high probe loading capacity and low degree of non-specific binding. For our experiments, we have chosen a surface developed by Harbers et al. (referred to as 3D hydrogel) [55]. The 3D network consists of a carbohydrate-based Tween derivative cross-linked with polyethylene glycol (PEG) and attached to the slide surface by an activated silane. PEG chains not used for crosslinking present NHS ester groups that allow for covalent probe immobilization.

The 2D epoxy and 3D hydrogel surfaces provide very different environments for oligonucleotide immobilization. The main difference is the presence of water in the hydrogel that provides favorable conditions to support immobilization. The two different types of slides can be considered representative for other 2D silane and 3D hydrogel surfaces.

In the following, we present the results of investigations on the immobilization of dye labeled 20mer oligonucleotides with (aACG) and without (ACG) a 3′-aminoheptyl linker on the surface of 2D epoxy and 3D hydrogel activated slides in different liquid volumes. The immobilization behavior in the microarray format (fast drying, nL case) is compared to liquid phase immobilization in a homogeneous solvent environment (no drying, liquid solution) and to pipetted arrays applying μL drop volumes (environmentally controlled, μL case). We show that besides the chemical reactivity of the substrate surface, the applied liquid volume controls the effectiveness of an activating amine linker for surface binding.

## Results and discussion

In the following we describe experiments performed to determine the dependence of the immobilization efficiency on probe concentration, immobilization time, and spotting buffer composition with respect to pH and ionic strength. As probes, two 5′ Cy3 labeled 20mer oligonucleotides of the same sequence (aACG, ACG) were used. In contrast to ACG, oligonucleotide aACG carries a 3′-aminoheptyl linker modification. The fluorescent Cy3 dye allows a direct observation of immobilization effects by applying a simple processing protocol consisting of only four experimental steps, i.e. probe deposition by piezo-electric spotting or manual pipetting, immobilization, washing, and slide drying. For nL and μL volumes applied in the array experiments, oligonucleotides were immobilized to the slide surfaces in a humidity chamber, followed by washing and drying in an automated hybridization station in order to reduce variability between measurements. For investigation of liquid phase immobilization, all protocol steps were performed in a hybridization station. In all cases, fluorescence scanner images were recorded and the fluorescence intensities analyzed. All experiments were performed under identical conditions with respect to buffer composition (150 mM, pH 8.5) and oligonucleotide concentration (2.5 – 20 μM), unless stated otherwise.

### Spotted microarrays: the nL case

In a typical microarray experiment, arrays are spotted with devices applying (sub-) nL volumes leading to spots with diameters in the order of 100 μm. Such small volumes of aqueous buffer solutions will inevitably evaporate within seconds unless additives are used to delay this process (see below).

**Figure 1:**
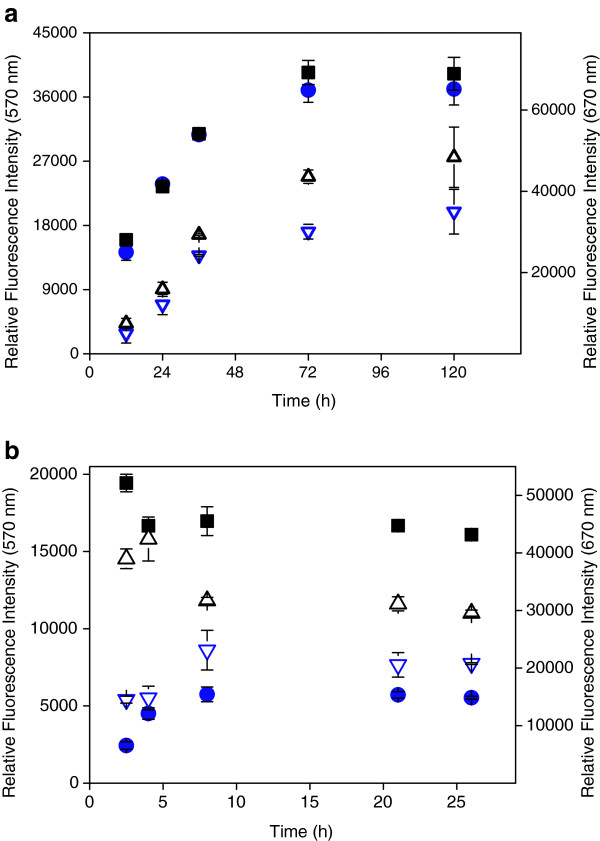
**Time dependence of oligonucleotide immobilization.** Dependence of averaged mean fluorescence intensity on immobilization time for aACG (black squares) and ACG (blue circles) at 20 μM spotting concentration (left scale). Averaged mean fluorescence intensity after 2 hours hybridization with 200 nM complementary Cy5 labeled oligonucleotide for aACG (black triangles) and ACG (blue triangles) under low-stringency conditions at 23°C (right scale, for experimental details see Methods section). (**a**) On 2D epoxy slides and (**b**) on 3D NHS hydrogel slide. Error bars that are not visible are hidden behind the symbols.

In the first experiment, we have investigated the time dependency of immobilization for dilution series of aACG and ACG on 2D epoxy and 3D hydrogel surface, respectively. In Figure 1a, the time course data are shown for the two oligonucleotides over a period of 5 days, spotted at a concentration of 20 μM. On the 2D epoxy surface, the amount of immobilized probe slowly increases with time until a plateau is reached after 72 hrs. A similar binding kinetics is observed for other oligonucleotide concentrations of the dilution series except for the 2.5 μM solutions that reach the plateau already after 36 h (data not shown). It is apparent that there are no differences between aACG and ACG. Obviously, under these conditions the presence of an aminoheptyl linker has no effect on immobilization.

In order to exclude an influence of the labeling dye, following immobilization we hybridized the slides at 23°C with the reverse complementary 5′ Cy5-labeled 20mer oligonucleotide and washed under non-stringent conditions at 23°C (triangles in Figure 1). For all spotted oligonucleotide solutions, the time courses of fluorescence measured at 670 nm (Cy5) follow that of immobilization at 570 nm (Cy3). This proves that functional immobilization indeed continues until a maximum is reached after about 3 days. The matching curves also prove that oligonucleotides are immobilized in a way that they are accessible for hybridization, as it was previously shown in a similar experiment [57].

On the 3D hydrogel surface, immobilization is much faster (Figure 1b). aACG and ACG reach an intensity maximum already after 2.5 and 8 hrs, respectively. Since the spotting procedure took about half an hour, the first data point at 2.5 hrs is prone to a relatively large experimental error on the time scale. For this reason, we did not investigate immobilization at shorter times.

In contrast to their identical behavior on 2D epoxy, aACG and ACG differ with respect to the maximal intensities on 3D hydrogel. aACG shows an approximately 3 to 8 times higher intensity on a 3D hydrogel surface. As for 2D epoxy, we hybridized these slides at 23°C with the reverse complementary 5′ Cy5-labeled 20mer oligonucleotide and washed under non-stringent conditions at 23°C (Figure 1b, triangles). The matching course of the curves for immobilization and hybridization confirms that increasing fluorescence intensities are related to an increasing number of immobilized oligonucleotide molecules. The same results were found for lower spotting concentrations. When washing is performed under stringent conditions at 60°C, we find a much larger intensity ratio of aACG and ACG on 3D hydrogel (14 – 22). Stringent washing removes Cy5 fluorescence nearly completely from immobilized ACG (data not shown) in agreement with results reported by Harbers et al. (see Figure six(a) in [55]). This observation indicates that ACG immobilization takes place via amine groups of the bases. Dufva, Grainger and others addressed the question of the reactive site of bond formation in unmodified oligonucleotides [58,59]. Millan et al. observed covalent immobilization of DNA to NHS-activated glassy carbon electrodes to occur predominantly through deoxyguanosine residues [60]. In this context, Ghosh stated that coupling through the bases potentially compromises the effectiveness of the immobilized nucleic acid in hybridization reactions [61]. This would explain how affected bases in ACG linked to the 3D hydrogel surface are not able to contribute to hybridization giving rise to decreasing melting temperature.

**Figure 2:**
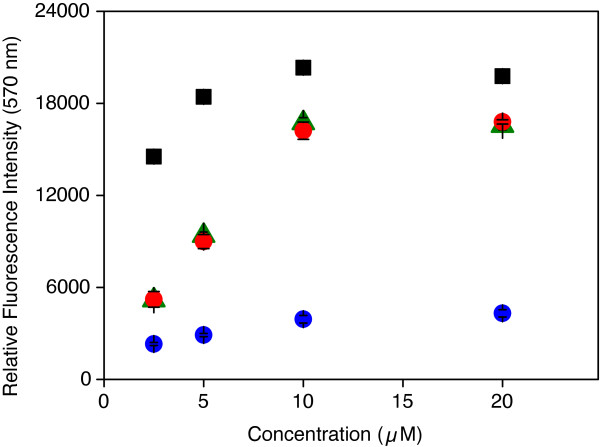
**Concentration dependence of oligonucleotide immobilization.** Averaged mean fluorescence intensity of dilution series after 18 hours of immobilization. On 2D epoxy: aACG (green triangles) and ACG (red circles). On 3D hydrogel: aACG (black squares) and ACG (blue circles). Error bars that are not visible are hidden behind the symbols.

It has to be stressed that by comparing fluorescence intensities on 2D epoxy and 3D hydrogel as shown in Figure 2 no conclusion can be drawn with respect to the absolute numbers of immobilized molecules on the two surfaces since local environmental conditions are very different. aACG and ACG immobilized on 2D epoxy are in dry state without a solvent present to stabilize the dye. On a 3D hydrogel surface oligonucleotides are located in an aqueous environment. Since dye fluorescence properties are known to depend on the local environment, the absence of solvent may have a significant effect on the fluorescence quantum yield [62]. The fluorescence quantum yield of Cy3-labeled oligonucleotides immobilized on a 3D hydrogel or 2D epoxy surface are not known, a fact that is often ignored when fluorescence intensities of immobilized dye labeled compounds are compared. For the same reason, care must be taken in interpreting fluorescence data from spotted dilution series in order to calculate the immobilization densities per area on a slide before and after washing.

Fluorescence intensities of dilution series for aACG and ACG on 2D epoxysilane and 3D hydrogel slides, respectively, after 18 h of immobilization are shown in Figure 2. This plot is of fundamental importance since it shows that the optimal spotting concentration for both surfaces is in the low μM range [33,63]. Increasing the concentration does not result in a higher number of immobilized oligonucleotides [64]. As for the time dependency, the concentration series data show no differences between aACG and ACG on 2D epoxy. Obviously, under conditions of nL spotting the reactivity of the two oligonucleotides is the same and the activating amine linker has no effect on immobilization. In contrast, on 3D hydrogel large differences for the two oligonucleotides are observed. Here, the amine linker activated oligonucleotide aACG shows about 5 times higher signal intensities than ACG at each concentration. At the other time points investigated, very similar results for both surfaces were obtained (data not shown).

These results show that there is a fundamental difference of the immobilization conditions on a 2D epoxy and a 3D hydrogel surface. A liquid volume of about 1 nL is typically spotted to the surface to generate a single spot. On both surfaces, the dispensed droplet will dry within a few seconds. Drop drying cannot be prevented even at very high ambient humidity because of the higher vapor pressure of a curved drop surface [22,65]. Therefore, on a 2D epoxy surface, time for probe immobilization via diffusive motion in the liquid drop is very short. Evaporation leaves the probe molecules in dry state incapable of significant molecular motions. Under these conditions, a reaction of the linker amine group can only occur when it is located in proximity to the surface. Considering the relative sizes of the 20mer oligonucleotide and the aminoheptyl linker it can be concluded that the larger the oligonucleotide or a probe in general is, the smaller is the chance for the amine linker group to react for sterical reason. With increasing molecular size, differences in reactivity of linker-modified and non-aminated oligonucleotides become negligible [58]. However, some minor movability must be remaining in a dried drop as indicated by the reported humidity dependence of immobilization [66] and the common practice to perform the immobilization step at high relative humidity. Reports on immobilization of highly activated oligonucleotides under drying drop conditions on the timescale of a few hours suggest that the reaction at the site of the linker group also proceeds in solid state [9,33,67].

The situation is different for 3D hydrogel. Under the spotting conditions of 50% relative humidity, the 3D polymer layer contains a high amount of water. On a hydrogel surface, conditions after deposition of a nL drop differ in two ways. First, at spotting a drop of about 90 μm diameter hits the surface and tends to penetrate the less than 100 nm thick hydrogel layer, and spreads. As for the 2D epoxy surface the drop will dry within seconds. However, even after drop drying is completed the thin hydrogel layer remains filled with solution. Within the hydrogel, probe molecules are able to diffuse and immobilize via molecular collisions comparable to conditions in liquid phase. Since the primary amine group of the linker is by far the most reactive site of the oligonucleotide it will preferably react with surface NHS groups at the polymer scaffold. This results in a much higher amount of probe immobilized via the linker compared to other molecular sites. However, comparing the relative intensities in Figure 2 the high amount of immobilized ACG is surprising considering the low reactivity of the nucleobases. This effect may be a caused by the unusual reaction conditions within the hydrogel (see following paragraph) and the number of 20 base amine groups present competing for reaction with one primary amine.

**Figure 3:**
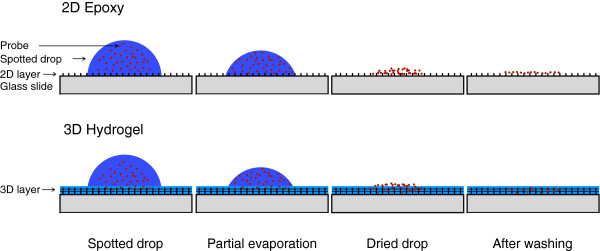
**Drop drying on 2D and 3D hydrogel surface.** Simplified scheme of drop drying on 2D epoxy and 3D NHS hydrogel slides, respectively.

The second effect is a concentration increase during drop drying. The evaporation of the drop on the surface leads to diffusion of probe and salt ions into the hydrogel layer driven by a rapidly increasing concentration gradient. The calculated volume of a spot is in the order of 1 pL, i.e. the volume within a spot surface area of 150 μm diameter and 100 nm height. Diffusion of all probe molecules from a nL drop into a pL spot volume would increase the concentration by a factor of 1000. We assume that the concentration increase is smaller considering the limited time and effects of associated increase in salt concentration and crystallization (“dehydration effect”). The increased concentration results in an increase of the reaction rate explaining the fast immobilization observed on the 3D hydrogel slides [68]. A high salt concentration also supports immobilization [59]. Under conditions preventing drop drying, the immobilization efficiency is reduced by a factor of more than 100. Drop drying and concentrating effect on 2D epoxy and 3D hydrogel after drop deposition are schematically illustrated in Figure 3.

**Figure 4:**
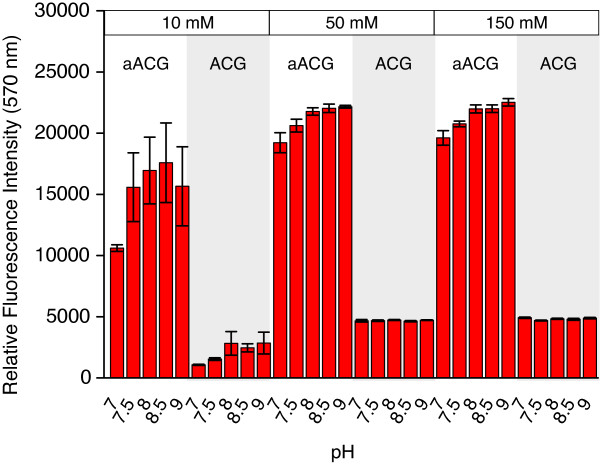
**pH dependence of oligonucleotide immobilization.** Influence of pH and buffer phosphate concentration on immobilization of aACG and ACG on 3D hydrogel after 22 hrs. Large error bars for aACG in 10 mM phosphate spotting buffer are due to poor spot morphology.

As mentioned above, the concentrating effect of a drying drop also affects the salt concentration. To investigate the influence of ionic strength and pH on the efficiency of oligonucleotide immobilization on the 3D hydrogel surface, the buffer composition was varied. According to the Henderson-Hasselbalch equation, drop volume changes are not expected to result in pH changes. The ionic strength, however, will increase with increasing salt concentration on drop drying caused by salt diffusion into the hydrogel layer. Arrays of 10 μM solutions of aACG and ACG, respectively, in 10 mM, 50 mM, and 150 mM phosphate buffer at pH 7 to 9 were spotted and processed after 4, 22, and 72 hrs as described in the Methods section (Figure 4).

As shown in the previous experiments (Figures 1 and 2), a higher immobilization efficiency was observed for the amine linker derivative aACG on 3D hydrogel. Surprisingly, there are only small effects on immobilization considering changes in proton concentration and ionic strength by a factor of 100 and (at least) 15, respectively (Figure 4). After 4 hrs, the immobilization is determined by differences in reactivity at a given pH and salt concentration (data not shown). Reactivity of primary amine groups increases with increasing pH in the investigated range [69]. Our data show that a high salt concentration supports immobilization and improves spot morphology. The excellent spot morphologies observed for 50 and 150 mM salt concentration are reflected by the very small standard deviations of the fluorescence intensities.

Immobilization is completed after 22 hrs. There are no differences in intensities measured at a later time point (72 hrs, data not shown). It is interesting to note that for 50 and 150 mM salt concentration, respectively, very similar fluorescence intensities were measured at pH 8 – 9. For ACG constant intensities are found. We assume that all available NHS ester groups have reacted. The competing side reaction, i.e. NHS ester hydrolysis, does not seem to play a role under the conditions applied. Our results confirm the specification of the slide manufacturer who recommends a phosphate concentration of 150 – 300 mM at pH 8.5 as optimal values for amine - NHS coupling.

### Immobilization in liquid phase

**Figure 5:**
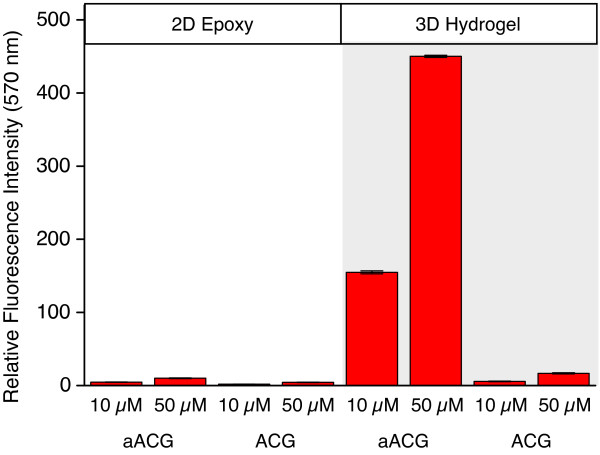
**Oligonucleotide immobilization in liquid solution.** Mean fluorescence intensities of liquid phase immobilization of aACG and ACG at concentrations of 10 and 50 μM, respectively, in 150 mM phosphate buffer after 20 hrs on 2D epoxy and on 3D NHS hydrogel slides. Measurements were performed with the same scanner settings as applied in the nL experiments to allow comparing intensities.

Immobilization experiments were performed under non-drying conditions in the sealed chambers of an automated hybridization station with sample volumes of 100 μL. We have studied the immobilization of 10 and 50 μM solutions of aACG and ACG, respectively, in 150 mM phosphate buffer at pH 8.5 on both types of slides. After 20 hrs, slides were washed under standard conditions and dried (see Methods section). Measured fluorescence intensities are about 100 to 1000 times lower compared to spotted arrays with nL drop volumes indicating a significantly lower immobilization efficiency in liquid phase (Figure 5). The reason for poor immobilization on 2D epoxy is the low probe concentration in combination with the relatively low reactivity of primary amines towards epoxy groups at pH 8.5. In case of spotting followed by drop drying, probe molecules are forced to a close contact with the surface. In contrast, under diffusive conditions in liquid phase repulsive interactions of the negatively charged glass surface with the oligonucleotide tend to reduce the number of molecular encounter. On the 3D hydrogel surface, amine reactivity to NHS ester is higher. Also, the influence of negative glass surface charges on immobilization is lower considering the hydrogel thickness of some 10 nm [55]. However, probe immobilization at low concentration has to compete with hydrolysis of NHS ester groups [61,70,71]. Compared to nL spotting experiments with increasing probe concentration on drop drying, the low fluorescence intensities observed for immobilization in liquid solution suggest that μM probe concentrations are too low for efficient immobilization [43,72]. There are only a few studies investigating oligonucleotide immobilization at higher concentration (for immobilization in non-drying μL drops that mimics liquid solution conditions see next paragraph). Gong et al. observed a similar but less pronounced effect on a polyacrylamide hydrogel surface (CodeLink) [59]. Comparing results of immobilization obtained by spotting and reaction in liquid solution, the authors found a 6fold reduction in intensity and a linear dependency of fluorescence intensity up to the highest probe concentration applied (400 μM). In one of the first studies on microarraying, Guo et al. observed increasing surface density on immobilization of an amine and oligo-T linker activated 15mer oligonucleotide at concentrations of 0.3 – 20 mM [25]. In following hybridization experiments, probe surface density obtained at a concentration of 5 mM was determined to result in the maximal hybridization signal. The low hybridization efficiency of 20% suggests that the oligonucleotide was immobilized not only via the amine linker but also via nucleobases.

An alternative to a high oligonucleotide concentration is the use of a more reactive coupling chemistry to reach efficient immobilization. There are many examples of such surface activation including maleimide [67,73,74], iso(thio)cyanate [25,75-77], thiol groups [33,78], and streptavidin [79]. Due to the high reactivity of these surfaces to thiol [67,73,75] and disulfide linker [33] activated and biotinylated [79] oligonucleotides, respectively, a much faster immobilization can be achieved within minutes to a few hours. E.g., Grunwell immobilized a 200 nM solution of a biotinylated oligonucleotide to a streptavidin coated surface within 15 min [79]. There is a simple relationship connecting the reactivity of a system of activating linker and surface and the specific site of immobilization. Since covalent side reactions, e.g. via bases are comparatively slow, the degree of immobilization via the linker increases with its reactivity to the surface. For highly reactive systems a high selectivity is observed, i.e. no immobilization of the non-activated oligonucleotide [33,37,73,80]. This ratio may be used to estimate the relative reactivity of a chosen coupling chemistry under the specific conditions at the surface.

We conclude that in liquid solution there is no efficient immobilization of both amine linker modified and non-aminated oligonucleotides at μM concentration onto both surfaces. The amount of oligonucleotide immobilized under these conditions is far too low to be useful in microarray applications.

### Arrays from pipetting: the μL case

In early studies on microarrays in the 1990s, spotting devices were not generally available and probes were applied by pipetting μL volumes. Typically, immobilization on 2D surfaces was investigated in drop volumes ranging from 0.5 to 5 μL oligonucleotide solutions at concentrations of 1 μM – 2 mM [25,27,32,37,59]. Immobilization was reported to proceed on a timescale between minutes and a few hours [27,37]. This seems to be in contrast to our results (Figure 1). However, in all reports on fast immobilization of amine linker activated oligonucleotides to 2D epoxy silane and 3D surfaces either the μL drop was allowed to dry (sometimes probably unintentionally), mM probe concentration or harsh reaction conditions were used [25,32,37,81].

The μL case is special for that it strongly depends on ambient relative humidity. First, the drying time increases extending the period the probe can react under diffusive conditions. Second, the rate of concentration increase on drying is decreasing. In a nL drop the highest concentration is reached within seconds giving the oligonucleotide only very limited time to react under diffusive conditions. In larger drops, a sufficiently high concentration may never be reached for a too slow drop evaporation. The borderline case of a non-drying drop is equivalent to the liquid phase immobilization we investigated above.

To our knowledge, there is no systematic study of oligonucleotide immobilization focusing on the differences between nL spotting and μL pipetting. For this reason and to fill the gap to liquid phase experiments, we have generated arrays by pipetting 1 μL volumes of 2 μM solutions of aACG and ACG, respectively, in 150 mM phosphate buffer at pH 8.5 to the 2D epoxy and 3D hydrogel surface.

At an ambient relative humidity of 20%, drops were drying on the timescale of minutes. To create non-drying conditions, a relative humidity of 100% was set in a home-made chamber containing a large volume of water. The slides were incubated in the dark for 10, 60 and 120 min, respectively, for both conditions. That drops were dried at 20% humidity and preserved at 100% humidity was confirmed by examination of the arrays using a microscope. Finally, slides were washed, dried and analyzed according to the standard protocol (see Methods section).

**Figure 6:**
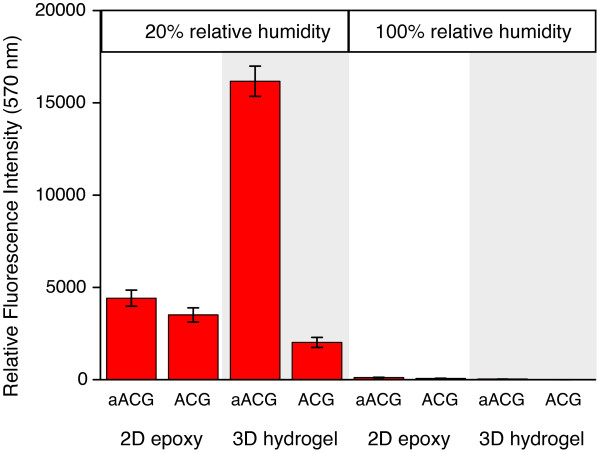
**Oligonucleotide immobilization in μL drops.** Humidity dependence. Mean fluorescence intensities after 120 min immobilization of 2 μM solutions of aACG and ACG on 2D epoxy and 3D hydrogel surface, respectively, in 1 μL drops. Slides were kept in the dark at 20% and 100% relative humidity. Measurements were performed with the same scanner settings as applied in the nL experiments to allow comparing intensities.

Figure 6 shows fluorescence intensities of aACG and ACG on the 2D epoxy and 3D hydrogel surface, immobilized in a 1 μL drop under drying and non-drying conditions, respectively. Immobilization on both surfaces only occurs at 20% relative humidity when drops are drying. In contrast, very small fluorescence intensity was observed for drops under non-drying conditions at 100% relative humidity.

When drops are allowed to dry on the 3D hydrogel surface, intense signals for aACG are observed already after 10 minutes. Conditions in the hydrogel are comparable to nL spotting where fast immobilization was observed due to the concentrating effect of the drying drop. aACG and ACG immobilization under drop drying conditions on 3D hydrogel is determined by the specific reactivity of molecular groups favoring the primary amine group of the linker.

On 2D epoxy, a 1 μL drop at 20% relative humidity dries within 10 min. During this period of time immobilization takes place under diffusive conditions preferring the reaction via the amine linker. As a result of slower drying, an increased probe concentration is present at the surface for minutes compared to seconds in nL spotting experiments. This strongly supports a fast immobilization and leads to a relatively high fluorescence intensity after short time (compare with first data point in Figure 1a). After drop evaporation, the probe is in dry state where significant molecular motion is prevented and immobilization is determined by the contact of the molecules with the reactive surface. After 120 min there is almost no difference between the immobilization efficiency of aACG and ACG. At very short times (10 min, data not shown), however, the intensity ratio of aACG and ACG is larger than two caused by the preferred immobilization of aACG under diffusive conditions. The immobilization behavior of both oligonucleotides under drying conditions correlates well with the results observed for nL spotting experiments shown in Figure 2.

At 100% relative humidity under non-drying conditions, a very low degree of immobilization was observed on both surfaces after 120 minutes (Figure 6). This confirms our results from the liquid phase experiments. Both oligonucleotides do not immobilize to a significant extent due to their low concentration and, additionally, the low reactivity in case of 2D epoxy. On 3D hydrogel, immobilization of probe at μM concentration cannot compete with hydrolysis of NHS ester groups. Though, there is a large difference for aACG immobilization between 2D epoxy and 3D hydrogel indicating the higher reactivity of NHS ester compared to epoxy (derived from comparing with intensities obtained in the dilution series experiments).

In an early study from 1994, Lamture et al. immobilized a 36mer oligonucleotide on 2D epoxy in a non-drying μL drop [37]. To ensure a sufficient reactivity of the amine linker, 0.1 M KOH solution was used as solvent. Under such harsh conditions, the primary amine group is reactive enough to yield sufficient immobilization compensating for the low probe concentration applied (5 – 50 μM, 6 hrs, 37°C). The authors also reported that the non-aminated oligonucleotide reacts only to a very low degree. From this publication and that of Guo et al. [25] the necessity of an activating amine linker was deduced although conditions at (sub-) nL spotting accompanied with fast drop drying are not comparable. These publications were cited more than 180 and 400 times, respectively.

The μL experiments also explain results of immobilization in the presence of additives in the spotting solution. Additives including betaine [82-84], formamide [85], and DMSO [63,83,84,86], are used to improve the efficiency of immobilization of dsDNA and oligonucleotides on positively charged surfaces coated with aminosilanes and poly-L-lysine, and to improve spot morphology. For unmodified dsDNA lacking reactive groups no stable covalent bonds can be formed. On surfaces containing amine groups, electrostatic interactions of the negative backbone with positive charges of surface amine groups lead to a sufficiently strong binding [87-89]. Drops of spotting solution containing the mentioned additives tend to dry much slower giving the DNA probe time to arrange at the surface under diffusive conditions in order to maximize binding. The reaction is driven by a high number of opposite charges compensating for the low DNA concentration that is typically applied.

In contrast, the use of additives has a negative effect for amine linker modified and non-aminated oligonucleotides, i.e. for probes of low reactivity, to be immobilized to on 2D epoxy via covalent bonds. Addition of betaine to the spotting solution leads to a partly drying drop giving rise to a relatively small increase of concentration [82], a situation similar to conditions in a μL drop at high relative humidity. We observed a low degree of immobilization of aACG and ACG on both 2D epoxy and 3D hydrogel, respectively, after 4 hrs in 150 mM phosphate spotting buffer at pH 8.5 containing 1.5 M betaine (data not shown).

To summarize, the results of experiments with μL drop volumes strongly depend on the relative humidity determining the rate of drop evaporation. Fast evaporation at low relative humidity leads to conditions comparable to spotting experiments (nL case). At high humidity, immobilization conditions in the drop closely resemble that in liquid solution.

## Conclusions

The fact that nL volume drops are quickly drying on a microarray surface is an essential precondition for oligonucleotide immobilization and, therefore, a key element for spotted microarrays to function. Due to the special conditions on drying, probe molecules are forced into close contact to the 2D epoxy coated glass surface that is negatively charged. The molecular contact supports a chemical reaction with the surface even under conditions that allow only marginal molecular motion. Preventing drop drying results in a low immobilization efficiency caused by the low reactivity of the amine - epoxy system at pH 8.5 and low oligonucleotide concentration. Diffusive conditions are a precondition for selective immobilization.

On the 3D hydrogel surface, drop drying leads to an increase of probe and salt concentration within the liquid phase of the hydrogel layer. In contrast to the 2D surface, reactions in the hydrogel always occur in liquid solution (unless it is intentionally dried) and are fast due to diffusive conditions and the reactivity of NHS ester groups to amines. Under such conditions, immobilization at the most reactive molecular site, i.e. the amine linker is preferred. As shown for ACG, immobilization also occurs to a remarkable extent via the bases. This is supported by the high number of available amine groups and the fact that reaction takes place at the polymer network and not directly at the negatively charged glass surface under the specific conditions within the hydrogel. As for 2D epoxy, without drop drying and a concomitant concentration increase there is no efficient immobilization, and a deactivating site reaction (NHS hydrolysis) controls proceedings.

The fact that the oligonucleotide spotting concentration has an efficiency maximum at about 10 μM is striking (Figure 2). In contrast, the optimal spotting concentration of peptides is about 500 times higher in the low mM range, as determined by the concentration dependence of immobilization. We suppose that the negative charges at the oligonucleotide backbone are responsible for this effect. The investigation of modified oligonucleotides having no backbone charges failed so far for the insolubility of the resulting compounds in aqueous spotting solution. To our opinion, the low maximal spotting concentration of oligonucleotides in concert with low reactivity is the reason for the influence of drop drying on immobilization.

In practice, the 2D epoxysilane coated surface is one of the most widely used surfaces for the production of oligonucleotide microarrays. Our study leads to two important conclusions. First, the immobilization of oligonucleotides to this surface is a rather unfavorable process considering the unspecific way and the long reaction times. Only by the effect of drop drying forcing the oligonucleotide to a close contact with the surface immobilization at μM spotting concentration reaction can occur. And second, an activating amine linker is of no use for its low reactivity to epoxy groups under the specific conditions on a dry microrray surface. The fast drying of a spotted nL drop shortens the available time for reaction in liquid phase to seconds. Only under diffusive conditions, the amine group is able to contribute to immobilization. However, irrespective of the disadvantages, 2D epoxy surfaces are a reasonable compromise considering the alternatives. Microarray surfaces activated with more reactive chemical groups including maleimide, thiol, or streptavidin did not gain acceptance for reason of stability, efforts on thiol-linker modified probe preparation, and price. Therefore, 2D epoxy surfaces are the slides of choice for the majority of oligonucleotide applications. In case of very short oligonucleotides, we recommend to immobilize probes via an amine linker to 3D hydrogel surfaces.

3D hydrogel surfaces are well suited for oligonucleotide microarraying in spite of higher costs for the slide and the amine modified oligonucleotides. Under conditions of fast drop drying (that are usually fulfilled), the low spotting concentration is compensated by the concentrating effect allowing a fast immobilization under diffusive conditions. Here, the activation of the oligonucleotide by an amine linker is indispensable. Even though non-aminated oligonucleotides immobilize as well the way of attachment prevents hybridization under stringent conditions.

The present study also provides guidance for the determination of immobilization conditions for an oligonucleotide at a surface of unknown reactivity, independent of the volume applied (nL spotting, μL pipetting, or liquid solution). Our results are not limited to the two surfaces investigated but are representative for a variety of surfaces having different reactive groups and architectures. As a general rule for all combinations, the determining factor is whether immobilization is diffusion controlled or takes place in a dry state. Table 1 summarizes the results of the immobilization study.

**Table 1:** Comparison of oligonucleotide immobilization on 2D and 3D surfaces

**Applied volume/conditions**	**Amine-linker**		**No amino-linker**	
	**2D epoxy**	**3D hydrogel**	**2D epoxy**	**3D hydrogel**
nL				
drying^a^	high	high	high	high
*μ*L				
drying	high	high	high	high
not drying	none	very low	none	very low
liquid phase	none	very low	none	very low

Comparison of immobilization efficiencies of oligonucleotides with and without amine linker on 2D epoxy and 3D hydrogel slides for arrays spotted from nL drop volumes, pipetting μL volumes, and in liquid phase for probe concentration in the low μM range.

^(a)^hydrogel layer remains wet after drop drying.

As a practical approach, the necessity of an activating linker can be evaluated by performing a dilution series of the probe and the respective time dependency when using a linker-activated and the corresponding non-activated dye labeled oligonucleotide as model compounds in a suitable (buffer) solution. A subsequent stringent hybridization with a complementary oligonucleotide confirms accessibility of the immobilized probe and verifies the selectivity of the molecular recognition (see Figures 1 and 2). In the course of assay development, this procedure can be carried out within a few days using no more than 15–20 slides allowing for a simple characterization of the immobilization system.

## Methods

### Materials

HPLC purified oligonucleotides were received from Microsynth. Both compounds, aACG and ACG, consist of 20 bases with the same sequence 5′-ACGACGGCCAGAGACGAACC-3′ and are both labeled with CY3 at 5′ end. The sequence was designed for non-self-complementarity and absence of secondary structures. Compound aACG has an additional aminoheptyl linker at 3′ end. The 5′ CY5-labeled reverse complementary sequence 5′-GGTTCGTCTCTGGCCGTCGT-3′ was used for hybridization. Microarrays slides were purchased from Schott-Nexterion (Slide E, Slide H), and stored as recommended. 20× saline-sodium citrate (SSC) solution, containing 300 mM sodium citrate and 3 M sodium chloride, sodium dodecylsulfate, sodium diphosphate, sodium hydroxide, and Tween 20 were supplied from Sigma-Aldrich. Ultra pure and filtered water (MilliQ, Millipore) was used for preparation of all solutions.

### Oligonucleotide spotting

Hydrogel slides (Schott Slide H) stored at -20°C were allowed to adjust to room temperature before opening the box to avoid condensation on slides. Hydrogel and epoxysilane slides (Schott Slide E) were given 15 minutes to adjust to ambient conditions before spotting. Dilution series of oligonucleotides (2.5 – 20 μM in 10, 50, and 150 mM sodium phosphate, pH 8.5) were spotted in 15 replicates (3 drops of 0.4 nL volume per spot) using a non-contact piezo spotter (sciFLEXARRAYER S11, Scienion) at 21°C and 50% relative humidity.

### Reaction of oligonucleotides in liquid phase

For immobilization of the oligonucleotides in liquid solution an automated hybridization station was used (HS4800, Tecan). Reactions were performed at 23°C. Slides were first washed with 150 mM phosphate buffer/0.1% Tween 20 pH 8.5 for 2 minutes to wet the surface before 100 μL of oligonucleotide solution (10 μM and 50 μM, respectively) in 150 mM phosphate buffer pH 8.5 was applied. The hybridization station allows efficient mixing during reaction of 20 h (set to “maximum”). Slides were washed at 23°C for 2× 3 minutes with 2× SSC/0.2% SDS buffer and 0.2x SSC/0.2% SDS, resp., and 3 minutes with 0.2× SSC. Slides were dried with nitrogen in the hybridization station at 30°C. According to the manufacturer, there is no adsorption of oligonucleotide to the polysulfone chamber material.

#### Reaction of oligonucleotides in μL drops

1 μL Drops of the oligonucleotides in 150 mM phosphate buffer at pH 8.5 were applied by using an Eppendorf pipette. Slides were stored for 10, 60 and 120 minute, resp., in a chamber at 20% (ambient) and 100% humidity (set by a large volume of distilled water) in the dark at 23°C. After immobilization, slides were washed in the hybridization station with SSC buffer according to the protocol given above, and dried.

### Immobilization and slide processing

For immobilization slides were stored at 23°C and 75% relative humidity for up to 5 days in a humidity chamber containing a saturated sodium chloride solution. Tests have shown that this was not necessary for hydrogel slides for the aqueous conditions within the gel. For washing, the automated hybridization station was used. Slides were washed with SSC buffer according to the protocol given above, and dried.

### Oligonucleotide hybridization

100 μL of a 200 nM solution of the CY5-labeled oligonucleotide in 5× SSC/0.1% SDS was hybridized for 2 h at 23°C to four replicate slides in the automated hybridization station. Slides were washed with SSC buffer according to the protocol given above at 23°C (non-stringent) and 60°C (stringent), resp., and dried.

### Fluorescence imaging and data evaluation

Slides were scanned immediately after processing using an LS400 confocal microarray scanner (Tecan) at a resolution of 10 μm. CY3 (CY5) fluorescence was excited at 543 (633) nm and detected at 570 (670) nm through a bandpass filter. To compare fluorescence intensities, slides of each experimental series were scanned under identical conditions with respect to photomultiplier voltage settings at 10 μm resolution. Relative fluorescence intensities were calculated using ArrayPro 4.5 software (Media Cybernetics). Trimmed mean background subtracted spot intensities from 15 spots were averaged. Background was very low (in the order of 10 counts) and determined in a ring of a width of two pixels outside the spot. For determination of time dependencies and dilution series of immobilization, spot intensities from 3 slides were averaged. To allow comparing relative fluorescence intensities spot size differences must be taken into account. For this reason, the same number of image pixel was used for intensity calculation by using a circle diameter smaller than that of the smallest spot. This procedure is justified for the nearly perfect spot morphology, i.e. the distribution of oligonucleotide within the spots. Error bars are very small and sometimes hidden within the symbols used in the Figures. For investigation of immobilization in liquid phase, one slide per concentration (10 μM, 50 μM) of each oligonucleotide was used. On these homogeneously coated slides the same grid used for evaluation of spotted slides was used for determination of fluorescence intensity. For comparison, the spot raw intensities in an area within 15 grid spot positions were evaluated. Evaluated data were processed for presentation using Origin 6.0 (OriginLab Corp.).

## Competing interests

The authors declare that they have no competing interest.

## Authors’ contributions

The manuscript was written through contributions of all authors. Microarray experiments were performed by JS and CA. Data were interpreted by JS and WW. All authors have given approval to the final version of the manuscript.
